# Systemic Antimicrobial Therapy for Diabetic Foot Infections: An Overview of Systematic Reviews

**DOI:** 10.3390/antibiotics12061041

**Published:** 2023-06-12

**Authors:** Angela Wright, Stephen Wood, Janath De Silva, J. Simon Bell

**Affiliations:** 1 Centre for Medicine Use and Safety, Faculty of Pharmacy and Pharmaceutical Sciences, Monash University, Melbourne, VIC 3052, Australia; 2 Pharmacy Department, Mackay Base Hospital, Mackay, QLD 4740, Australia; 3 Medicine Department, Mackay Base Hospital, Mackay, QLD 4740, Australia

**Keywords:** diabetic foot infection, systemic antibiotic treatment, diabetic foot ulcer, antimicrobial treatment, systematic review

## Abstract

Diabetic foot infections (DFIs) are a common complication of diabetes; however, there is clinical uncertainty regarding the optimal antimicrobial selection. The aim of this review was to critically evaluate the recent systematic reviews on the efficacy and safety of systemic (parenteral or oral) antimicrobials for DFI. Medline, Embase, CENTRAL, and CINAHL databases and the PROSPERO register were searched from January 2015 to January 2023. Systematic reviews with or without meta-analyses on systemic antimicrobials for DFI, with outcomes of clinical infection resolution or complications, were included. Of the 413 records identified, 6 systematic reviews of 29 individual studies were included. Heterogeneity of individual studies precluded meta-analysis, except for ertapenem versus piperacillin–tazobactam (RR 1.07, 95% CI [0.96–1.19]) and fluoroquinolones versus piperacillin–tazobactam (RR 1.03, 95% CI [0.89–1.20]) in one review. The application of the AMSTAR-2 tool determined two reviews to be of high quality. There was no statistical difference in the clinical resolution of infections for 24 different antimicrobial regimens (penicillins, cephalosporins, carbapenems, fluoroquinolones, vancomycin, metronidazole, clindamycin, linezolid, daptomycin, and tigecycline). However, tigecycline did not meet non-inferiority against ertapenem ± vancomycin (absolute difference −5.5%, 95% CI [−11.0–0.1]) and was associated with a higher incidence of adverse drug events. There is minimal systematic review evidence to suggest one regimen is superior to another for DFI.

## 1. Introduction

Diabetic foot infection (DFI) is a common complication of diabetes; however, there is clinical uncertainty regarding the optimal antimicrobial selection. DFI comprises skin and soft tissue infections (SSTIs) or extends into the bone as diabetic foot osteomyelitis (DFO) [1]. Effective treatment reduces the outcome of lower limb amputation and improves quality of life [1,2]. Acute versus chronic infections are associated with different microorganisms, with polymicrobial infections more likely in chronic ulcers [3,4]. Most guidelines recommend Gram-positive coverage alone for mild DFI; however, the international guidelines differ with regard to treating moderate-to-severe infections [1,3,5]. The most recent International Working Group on the Diabetic Foot (IWGDF) guideline recommends moderate-to-severe DFI coverage extends to Gram-negative and potentially anaerobes and pseudomonal cover based on patient factors [1]. It suggests classes of agents to consider, but does not provide a specific empiric regimen [1]. The National Institute for Health and Care Excellence (NICE) guidance does recommend specific antibiotics for moderate-to-severe DFI, with extended cover including gentamicin, metronidazole, amoxicillin–clavulanate or co-trimoxazole [5]. *Pseudomonas* and methicillin-resistant *S. aureus* (MRSA) cover are recommended if these are suspected or confirmed [5]. The archived IDSA guideline from 2012 suggests broad-spectrum therapy for severe DFI, but provides no recommendations for antimicrobial classes or specific agents [6]. These broad recommendations may reflect the lack of high-quality studies comparing specific antimicrobial treatments or may reflect different interpretations of the available evidence.

The objective of this study was to critically evaluate the recent systematic reviews of systemic antimicrobial treatments for treating DFI, with a focus on the clinical resolution of infection, complications, and adverse effects. An overview of the systematic reviews was conducted because multiple systematic reviews on the treatment of DFI have been published since 2015 and have not provided firm treatment recommendations. This overview aims to summarize the current systematic review evidence base available on this topic to guide clinical practice. An assessment of the methodological quality of these reviews and a summary of the outcomes are needed to inform clinical practice.

## 2. Methods

The overview of systematic reviews was conducted using the methodology described by Smith et al. [7] and the Cochrane Handbook, chapter on Overviews of Reviews [8]. The overview was reported in accordance with the Preferred Reporting Items for Systematic Reviews and Meta-Analyses (PRISMA) Statement [9], and the search was updated as described by Bramer et al. [10]. 

### 2.1. Search Strategy

Medline, Embase, CENTRAL, and CINAHL databases and the PROSPERO register were searched up to January 2023. The PROSPERO register of systematic reviews was searched to locate protocols of potentially relevant systematic reviews not identified through the biographic databases. The reference lists of included reviews were additionally screened to identify any further reviews for inclusion that may have been missed in the database search. 

The Medline search consisted of the Medical Subject Heading (MeSH) terms Diabetic Foot/, Foot Ulcer/, Soft Tissue Infection/ with keyword searching of “diabet*” close to “(foot or feet)”, “diabet*” close to “ulcer”, or “diabet*” close to “wound”. This was combined using Boolean operator AND with MeSH Antibiotic Agent/, Anti-infective Agents/, and the keywords “Antibiotic*” and “Antimicrobial*”. The search was limited to English language articles and meta-analysis or systematic reviews. The full search strategy for each database is detailed in Appendix A. 

### 2.2. Selection Criteria

Systematic reviews with or without meta-analysis published from January 2015 to January 2023 analyzing either randomized controlled trials (RCTs) or observational studies were included. This timeframe was chosen because the most recent Cochrane Database of Systematic Review on this topic was published in 2015, and multiple systematic reviews have been published since then [11]. The original studies within the reviews had no date exclusion. For the purpose of this overview, a systematic review was defined as utilizing a comprehensive, documented, and repeatable process to search for studies and assessment of quality and outcomes to answer a defined question. Systematic reviews of studies conducted for adults aged 18 years or over with diabetes of any type and receiving treatment for DFI were considered the population. Eligible reviews included studies with either or both SSTI of the foot or DFO. Eligible reviews evaluated systemic (parenteral and/or oral) antimicrobials for the treatment of DFI as the primary intervention. Comparators could include another systemic antimicrobial or topical treatments, wound care, or placebo. The primary outcomes included clinical resolution or improvement of infection, microbiological eradication, recurrence of infection, rehospitalization, amputation, or mortality. The secondary outcome was adverse drug events. If a review reported treatment of DFI in people with and without diabetes, results for people with diabetes had to be separable. Systematic reviews that examined other treatments in addition to systemic antimicrobials were included if the data for systemic antimicrobials were separable. Reviews focused solely on topical antimicrobials or other topical treatments, surgical interventions, or wound care were excluded.

### 2.3. Data Collection and Extraction

EndNote 20 was used to manage citations from the database search and assist with removal of duplicates and articles published prior to 2015. Two investigators screened the titles and abstracts of remaining citations to exclude those not relevant to the research question. Full text articles were then retrieved to assess suitability as per the inclusion criteria. The PRISMA flow diagram was used to document this process [9]. The eligibility of articles for inclusion and data extraction was undertaken independently by two investigators. Outcomes were assessed as reported in the systematic reviews; the primary study data were not re-analyzed in this overview. If the systematic reviews included outcomes that were not applicable to this overview, only the relevant outcome data were extracted.

### 2.4. Quality Assessment

The AMSTAR-2 (A measurement tool to assess systematic reviews) was utilized to review the methodological quality of the included systematic reviews [12]. Each systematic review was independently assessed by two investigators against 16 criteria, with a consensus obtained on any variances. From this assessment, each review was given an overall confidence rating; however, it was not intended to calculate a total score.

## 3. Results

### 3.1. Summary of Included Systematic Reviews

**Figure 1:**
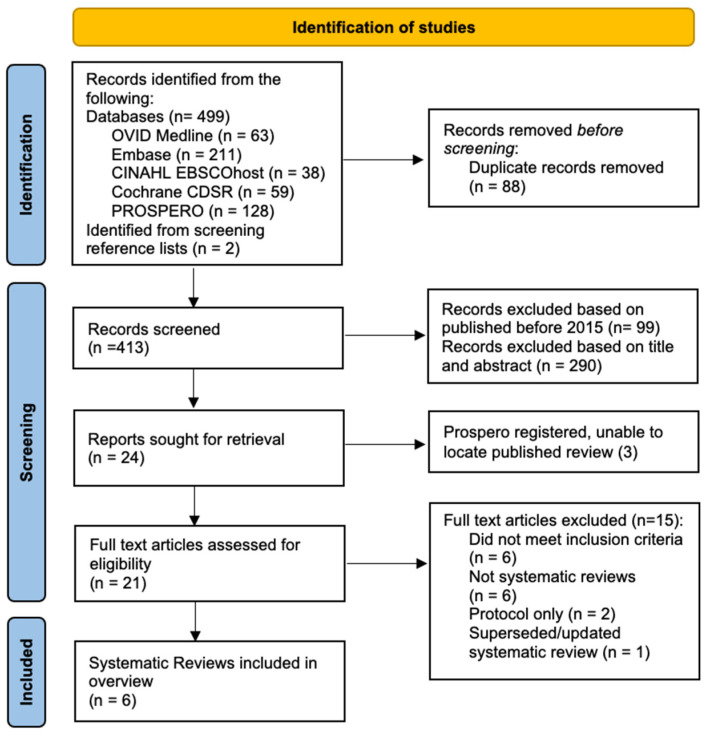
PRSIMA flow chart.

A total of 499 records were extracted from databases and 2 from screening reference lists. After duplicate removal, 413 articles were screened based on the year published, and then the title/abstract was reviewed against the inclusion criteria. The full text of 21 articles was assessed, with six systematic reviews meeting the criteria for inclusion (Figure 1) and reasons for exclusion detailed in Appendix B. 

**Figure 2:**
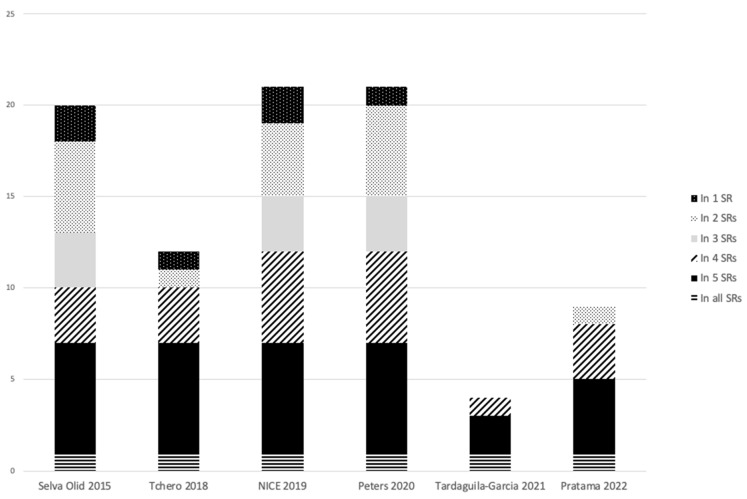
Overlap of included studies in systematic reviews [11,13,14,15,16,17].

Six systematic reviews with twenty-eight individual RCTs and one cohort study were included and are described in Table 1. Five systematic reviews included RCTs only, and one included RCTs and observational studies. Of the 29 individual studies included in the systematic reviews, 4 were published within the last 10 years, and the year published ranged from 1983 to 2016 [11,13,14,15,16,17]. The 2015 Cochrane review included RCTs investigating systemic antimicrobials for DFI [11]. We also included a 2018 systematic review of open and blinded RCTs of systemic and topical antibiotics [15]. Two of the systematic reviews included were performed to enable the development of guidelines: the IWGDF guideline and the NICE guideline on diabetic foot problems (NG19) [1,5,13,14]. We also included a 2021 review that was focused on medical and surgical treatment of DFO, and lastly, a 2022 review of RCTs assessing systemic and topical antibiotics in addition to microbiological profiles [16,17]. Two of the systematic reviews updated primary data to produce risk ratios and 95% confidence intervals where possible [11,13]. Patients with osteomyelitis (OM) were excluded from 11 studies in the Cochrane review, included in 11 studies in the Peters et al. review, and were the sole focus of the Tardaguila-Garcia et al. review [11,14,16]. The Cochrane review included two individual studies with male participants only, one study did not provide data, and the remaining studies had an average of 62% male participants with a mean age of 61.4 years [11]. Figure 2 demonstrates the overlap of the studies included in the reviews. There was one study included in all six systematic reviews, six studies included in five of the reviews, and a further five studies were included in four reviews. There were only 6 studies included in a single systematic review. The most recent review published in 2022 contained no studies that were not included in any prior systematic reviews [17].

### 3.2. Quality of the Systematic Reviews

The results of the AMSTAR-2 assessment are detailed in Table 2. All reviews provided the PICO components, assessed the risk of bias for the included studies, considered the risk of bias when discussing the study results, and reported the potential conflicts of interest of the review authors. Funding for the included studies however was not reported in three cases. Four reviews did not justify the exclusion of individual studies, which is a critical weakness as per AMSTAR-2, resulting in a lower confidence rating [12]. The Cochrane and NICE reviews achieved high confidence ratings containing almost all components of the AMSTAR-2, excluding an explanation for only including RCT study designs. The Cochrane review was the only systematic review to perform a meta-analysis, with the consideration of individual study bias and investigation of publication bias completed [11].

### 3.3. Outcomes: Antimicrobial Interventions

The antimicrobial regimens investigated in the six systematic reviews included penicillins, cephalosporins, carbapenems, fluoroquinolones, vancomycin, metronidazole, clindamycin, linezolid, daptomycin, and tigecycline [11,13,14,15,16,17]. The 24 different regimens along with the effect estimates as reported in the systematic reviews are detailed in Table 3. Heterogeneity of the regimens and outcomes and the high risk of bias from the lack of blinding precluded the meta-analysis for the most part [11]. The meta-analysis was performed in the Cochrane review to analyze ertapenem versus piperacillin–tazobactam and fluoroquinolones versus piperacillin–tazobactam/amoxicillin–clavulanate [11]. For most of the antimicrobial regimens compared in the systematic reviews, there was no statistical difference in the clinical resolution of infections. For some of the higher-quality studies, evidence for adverse drug events and variability in reporting are detailed below.

#### 3.3.1. Fluoroquinolones versus Piperacillin–Tazobactam (TZP)/Amoxicillin–Clavulanic Acid (AMC)

A study by Schaper et al. [19] included in five out of the six systematic reviews found similar outcomes in the clinical resolution and complications between moxifloxacin and TZP/AMC with RR 0.98 (95% CI [0.84–1.13]) [13]. Peters et al. described this study as high quality; however, the NICE review scored it as moderate quality [13,14]. A pooled analysis of three studies (387 participants) using a fixed effect model (I^2^ = 0%) to compare fluoroquinolones against TZP found no significant difference between the two treatments (RR 1.03, 95% CI [0.89–1.20]) [11].

#### 3.3.2. Ertapenem (ETP) versus TZP

Three studies included in the reviews assessed ETP against TZP ± vancomycin (Xu et al. [25], Graham et al. [26], Lipsky et al. [27]). Xu et al. conducted a non-inferiority study, which demonstrated no significant differences in the outcomes as per the NICE review [13]. In a subset analysis of severe DFI, ETP had a lower rate of resolution, with Tchero et al. reporting this as significant [15]. Peters et al. discussed this non-inferiority trial, asserting that it was not powered to determine whether a statistically significant difference exists [14]. The other studies demonstrated no statistically significant differences [26,27]. Two studies (684 participants) were pooled in the Cochrane review (Graham et al. [26], Lipsky et al. [27]) using a random effects model (I^2^ = 0%) and found no difference between the treatment groups (RR 1.07, 95% CI [0.96–1.19]) [11].

#### 3.3.3. Tigecycline (TGC) versus Ertapenem (ETP) ± Vancomycin (VAN)

An RCT by Lauf et al. [45] included in all the systematic reviews concluded that TGC did not meet non-inferiority against ETP ± VAN (absolute difference −5.5%, 95% CI [−11.0–0.1]) for the clinically evaluable population. TGC was also associated with a higher incidence of adverse events (nausea, vomiting, and insomnia) [13,14]. The NICE review analysis of this study reported no significant difference in clinical cures (RR 0.94, 95% CI [0.99–1.14]) [13]. It scored the study as moderate-quality evidence with a serious risk of bias [13]. The Cochrane review conversely reported that this non-inferiority study of ETP ± VAN resulted in higher rates of clinical resolution (RR 1.09, 95% CI [1.01–1.18]) with low risk of bias [11]. In the osteomyelitis subgroup, the Cochrane review reported higher rates of resolution with ETP ± VAN (RR 2.08, 95% CI [1.27–3.39]) [11]. This OM subset was not planned to have a statistical analysis in the original trial [45].

#### 3.3.4. Ampicillin–Sulbactam (SAM) versus Cefoxitin (FOX)

A small double-blind study by Erstad et al. [34] found non-significant lower resolution rates with SAM compared with FOX (RR 0.14, 95% CI [0.02–1.05]) [11]. In a subset of patients with osteomyelitis from this same study, Peters et al. reported a higher cure rate with cefoxitin [14]. The study was underpowered with 36 participants, had low overall resolution rates compared with other studies, and has a short duration of treatment of 6 days [14].

#### 3.3.5. Adverse Drug Events

Incidences of nausea, vomiting, and insomnia were higher with TGC compared to ETP ± VAN (RR 1.25, 95% CI [1.13–1.38]) [13,14]. The NICE review reported moderate-quality evidence for higher adverse events (diarrhea, nausea, and anemia) with linezolid compared with SAM/AMC (RR 2.66, 95% CI [1.49–4.73]) [13]. The NICE review reported that moxifloxacin demonstrated a higher rate of adverse events than TZP/AMC (RR 2.54, 95% CI [1.21–5.34]) but not study withdrawals [13]. The Cochrane review reported a lower risk of adverse events with daptomycin than vancomycin or semi-synthetic penicillin (RR 0.61, 95% CI [0.39–0.94]) [11]. One trial (Saltoglu et al. [30]) included in five of the six systematic reviews was described by Cochrane as showing a non-significant higher rate of adverse events with TZP versus imipenem–cilastatin (IMP) (RR 3.19, 95% CI [0.95–10.72]) and more cases of hepatotoxicity/nephrotoxicity [11]. The NICE review reported no significant difference, while the other reviews did not discuss adverse events for this study [13]. 

## 4. Discussion

This overview of the systematic reviews on parenteral and oral antimicrobials in DFI identified six reviews published between 2015 and 2022 [11,13,14,15,16,17]. A Cochrane review was the only review to perform a meta-analysis; two reviews were conducted to inform guideline development; and one review focused on DFO [11,13,14,15,16,17]. Overall, the reviews were of variable quality as per the AMSTAR-2 tool, with the Cochrane and NICE systematic reviews having the highest confidence ratings. Considerable overlap in the included studies was identified in Figure 2. The reviews incorporated RCTs, except for one cohort study relevant to this review incorporated by Peters et al. [14]. Considering a broader range of observational studies would be unlikely to add value regarding efficacy, 29 studies were already included within the reviews. The heterogeneity of studies with different antimicrobial regimens, definitions for DFI and DFO, and duration of treatment made it difficult for the systematic reviews to pool data for meta-analysis and provide clear evidence for specific regimens. Despite this, the evidence base does demonstrate consistent findings for equivalence for most systemic antibiotic regimens studied for DFI, with no one regimen superior.

The results of the AMSTAR-2 assessment in Table 2 show a variable level of confidence in the methodological quality of the systematic reviews performed. The Cochrane and NICE reviews were assessed as high confidence, with the only non-critical weakness being a lack of explanation for only including RCTs [11,13]. The other four reviews achieved a lower confidence rating, owing to the critical weakness of not providing a list of excluded studies with the reasons for exclusion. Peters et al. [14] received a moderate confidence rating, as detailing the excluded reviews was deemed impractical with a high quantity. Tchero et al. [15], Tardaguila-Garcia et al. [16], and Pratama et al. [17] did not state the funding source for the included studies to allow for a comprehensive assessment of bias. Peters et al. [14] included a discussion on the quality of the included studies and highlighted the higher-quality study outcomes. The Cochrane and NICE reviews included comprehensive outcome data on all included reviews, including calculating risk ratios and confidence intervals for data where appropriate [11,13]. Future systematic reviews could improve their quality by utilizing the AMSTAR-2 tool for guidance [12].

### 4.1. Antibiotic Outcomes

Peters et al. [14] interpret the interventions as broadly equivalent, except for TGC versus ETP. The overall conclusions for all the interventions reported in the Cochrane review and the NICE review are similar [11,13]. When comparing fluoroquinolones with TZP/AMC, Peters et al. [14] graded Schaper et al. [19] as high quality, whereas the NICE review graded it as moderate quality [13]. Cochrane assessed this study as having an unknown risk of bias in two domains, which could account for this variance; the pooled analysis found no significant difference between fluoroquinolones and TZP/AMC [11]. The study by Xu et al. [25] comparing ETP versus TZP in a severe DFI subgroup is described by Tchero et al. [15] as demonstrating significant outcomes. In contrast, Peters et al. [14] describe this trial as a non-inferiority study, designed to demonstrate the equivalence and not the superiority of a regimen. In addition, the pooled analysis for ETP versus TZP in the Cochrane review found no significant difference between the groups [11]. TGC did not meet non-inferiority to ETP in a trial by Lauf et al. [45], which was consistently interpreted in the systematic reviews. Differences in the result values reported for this study between reviews are due to NICE reporting as per the clinically evaluable (CE) participants, whereas the Cochrane review is as per the randomized intention-to-treat groups (ITT) [11,13]. Cochrane graded this evidence at a low risk of bias, differing from the NICE evidence summary, which graded it as moderate quality with a serious risk of bias [11,13]. Of all the antibiotics reviewed, there was no difference between regimens with or without pseudomonal cover or between those with Gram-positive cover and additional Gram-negative or anaerobe cover.

The Cochrane review described the evidence for adverse events between treatments as unclear; however, there were some interesting findings regarding the safety of antibiotic regimens for DFI [11]. The systematic reviews included found a higher likelihood of adverse events with TGC compared with ETP (± VAN) [11,13]. Linezolid was more likely to cause adverse events compared with a broad-spectrum penicillin, and vancomycin or a semi-synthetic penicillin were more likely to cause adverse events than daptomycin [11,13]. Interestingly, the Cochrane review highlighted a non-significant difference in adverse events between TZP and IMP from Saltoglu et al. [11,30]. The NICE summary described no difference in adverse events between these treatment groups [13].

### 4.2. Strengths and Limitations

This overview of systematic reviews assessed six recent systematic reviews, including twenty-nine individual studies, summarizing twenty-four different antimicrobial regimens. A strength was the use of the AMSTAR-2 tool to critically assess the quality of the included reviews, which was independently performed by two investigators. A limitation of this overview was restricting the search to the English language. Although a comprehensive literature search of bibliographical databases was completed, the gray literature search was limited to the Prospero register, searching guidelines, and reference lists. The primary focus was on systemic antimicrobials (parenteral and oral), with the literature on topical and other non-antimicrobial therapies excluded. No meta-analysis was conducted due to the heterogeneity of the reviews included.

## 5. Conclusions

There is minimal systematic review evidence to suggest that one antimicrobial regimen is superior to another for diabetic foot infections. Tigecycline failed to meet non-inferiority compared to ertapenem ± vancomycin, and a higher risk of adverse drug events was associated with tigecycline and linezolid. High-quality studies with adequate concealment to reduce the risk of bias are needed to improve the evidence base. Further research should involve both randomized controlled trials of new therapeutic regimens (with less risk of bias) and high-quality observational studies (to assist in identifying particular patient groups or organisms for which current regimens may have superior efficacy). Another relevant issue is a comparison of the effectiveness of an early stepdown from intravenous to oral antimicrobials in moderate to severe DFI. Future systematic reviews on this topic could be improved by utilizing the AMSTAR-2 tool.

## Figures and Tables

**Table 1:** Summary of the characteristics of included systematic reviews.

	Types/# Studies	PICO Question/s	Comments
Selva Olid et al. 2015 (Cochrane Review) [11]	Twenty RCTs	P—T1DM/T2DM with foot infection. I—Systemic antibiotics (oral or parenteral). C—Any other antibiotic control group, placebo or topical foot care. O—Resolution of infection, time to resolution, adverse effects, and complications.	Meta-analysis on two interventions: ETP vs. TZP (two studies) and fluoroquinolones vs. TZP/AMC (three studies). Uses Cochrane Risk of Bias tool.
Tchero et al. 2018 [15]	Twelve RCTs—open label and blind (relevant to this review)	P—T1DM/T2DM moderate–severe DFI ± OM, >18. I—All antimicrobials (including topical). C—Other antimicrobials, placebo, foot care, and surgery. O—Clinical cure or improvement rate.	Solely people with diabetes with complicated DFI. No meta-analysis. Uses Cochrane Risk of Bias tool.
NICE 2019 [13]	Twenty-two RCTs	P—≥72 h old, T1DM/T2DM, and foot ulcer with SSTI ± OM. I—Any antibiotic regimen. C—Standard care, other antimicrobials, and other treatments (not surgery). O—Cure rate, amputation, adverse events, length of stay, and QOL.	Completed to inform the NICE guideline. Children/young people in inclusion criteria; nil studies found. Recalculated results for studies presenting RR, 95% CI, and absolute differences. Uses GRADE assessment for risk of bias and quality.
Peters et al. 2020 (IWGDF) [14]	Twenty RCTs; one cohort study (relevant to this review)	P—T1DM/T2DM, ≥18. I—Antibiotics, antiseptic, surgery, and adjunctive therapy. C—Another intervention, placebo, no treatment, or usual care. O—Clinical cure of infection, amputation, recurrence, death, hospitalization, resolution of ulcer, eradication of microbial pathogens, QOL, adverse effects, or cost of treatment.	Completed to inform the IWGDF guideline. Analyzed SSTI and DFO separately. No meta-analysis. Uses Dutch Cochrane quality tool and SIGN level of evidence tool.
Tardaguila-Garcia et al. 2021 [16]	Four RCTs (relevant to this review)	P—>18 with DFO. I—Antibiotics or surgery. C—Antibiotics or surgery. O—Cure rates.	Focused on diabetic foot osteomyelitis. No meta-analysis Uses Cochrane Risk of Bias tool
Pratama et al. 2022 [17]	Nine RCTs (relevant to this review)	P—T1DM/T2DM, DFUI with/without OM, ≥18. I—Antibiotics (parenteral, oral, and topical). C—Other antibiotics or placebo. O—Clinical cure.	Additionally, reported microbiological profiles. No meta-analysis. Uses Jadad criteria for risk of bias and quality.

RCTs: randomized controlled trials; T1DM: Type 1 diabetes mellitus; T2DM: Type 2 diabetes mellitus; ETP: ertapenem; TZP: piperacillin–tazobactam; AMC: amoxicillin–clavulanate; SAM: ampicillin–sulbactam; OM: osteomyelitis; DFO: diabetic foot osteomyelitis; SSTI: skin and soft tissue infection; QOL: quality of life; DFUI: diabetic foot ulcer infection.

**Table 2:** Methodological quality of included systematic reviews using AMSTAR-2 tool.

AMSTAR-2 Items	Selva Olid 2015 [11]	Tchero 2018 [15]	Peters 2020 [14]	NICE 2019 [13]	Tardaguila-Garcia 2021 [16]	Pratama 2022 [17]
1. PICO components	Yes	Yes	Yes	Yes	Yes	Yes
2. A priori design	Yes	Partial	Partial	Yes	Partial	Partial
3. Explanation of study design inclusion	No	No	No	No	No	No
4. Comprehensive literature search	Yes	Yes	Yes	Yes	Partial	Partial
5. Duplicate study selection	Yes	Yes	Yes	Yes	Yes	Yes
6. Duplicate data extraction	Yes	Yes	Yes	Yes	Yes	Yes
7. List excluded studies	Yes	No	No	Yes	No	No
8. Characteristics of included studies	Yes	Partial	Yes	Yes	Partial	Partial
9. Report quality of included studies	Yes	Yes	Yes	Yes	Yes	Partial
10. Report funding of included studies	Yes	No	Yes	Yes	No	No
11. Appropriate meta-analysis method	Yes	n/a	n/a	n/a	n/a	n/a
12. Assess risk of bias on meta-analysis	Yes	n/a	n/a	n/a	n/a	n/a
13. Consider risk of bias with results	Yes	Yes	Yes	Yes	Yes	Yes
14. Consider heterogeneity with results	Yes	Yes	Yes	Yes	Yes	Yes
15. Assess publication bias	Yes	n/a	n/a	n/a	n/a	n/a
16. State conflicts of interest	Yes	Yes	Yes	Yes	Yes	Yes
Overall Confidence	High	Low	Moderate	High	Low	Low

**Table 3:** Outcomes of antimicrobial regimens from included systematic reviews.

Intervention	Comparator	Study ID	Outcome	Measure of Effect (95% CI) ^#^	Direction of Effect	SR Inclusion
**Fluoroquinolones**						
Moxifloxacin	TZP/AMC	Giordano 2005 [18]	Clinical resolution	RR 1.11 (0.80–1.54) [11]	NS	Selva Olid 2015 [11]
		Schaper 2013 (RELIEF) [19]	Clinical resolution Amputations Adverse events	RR 0.98 (0.84–1.13) RR 0.84 (0.51–1.38) RR 0.97 (0.66–1.42) [13]	NS NS NS	Selva Olid 2015 [11]; Tchero 2018 [15]; Peters 2020 [14]; NICE 2019 [13]; Pratama 2022 [17]
		Lipsky 2007 [20]	Clinical cure Adverse events Withdrawals	RR 1.14 (0.75–1.72) RR 2.54 (1.21–5.34) RR 1.02 (0.52–1.90) [13]	NS Favors TZP NS	Tchero 2018 [15]; Peters 2020 [14]; NICE 2019 [13]; Pratama 2022 [17]
Clinafloxacin	TZP/AMC	Siami 2001 [21]	Clinical resolution	RR 1.01 (0.55–1.86) [11]	NS	Selva Olid 2015 [11]; Peters 2020 [14]; NICE 2019 [13]
**Meta-analysis** Fluoroquinolones	TZP/AMC	Giordano 2005; Schaper 2013; Siami 2001	Clinical resolution	RR 1.03 (0.89–1.20) [11]	NS	Selva Olid 2015 [11]
Levofloxacin	Ticarcillin–clavulanate	Graham 2002b [22]	Clinical resolution	RR 0.97 (0.60–1.55) [11]	NS	Selva Olid 2015 [11]; Peters 2020 [14]
Moxifloxacin	AMC	Vick-Fragoso 2009 (STIC) [23]	clinical cure	RR 0.79 (0.57–1.08) [11]	NS	Selva Olid 2015 [11]; NICE 2019 [13]; Peters 2020 [14]; Pratama 2022 [17]
Ofloxacin	SAM/AMC	Lipsky 1997 [24]	Clinical resolution Amputation Adverse events	RR 1.13 (0.88–1.47) RR 0.11 (0.01–1.94) RR 1.82 (0.89–3.72) [11]	NS NS NS	Selva Olid 2015 [11]; Tchero 2018 [15]; NICE 2019 [13]; Peters 2020 [14]; Tardaguila-Garcia 2021 [16]
**Carbapenems**						
Ertapenem	Piperacillin–tazobactam	Xu 2016 ‡ [25]	Cure rate Cure (severe DFI) Clinical resolution Adverse events	Diff: −3.8% (−8.3–0.0%) Diff: –5.7% (−12.1–−0.3%) [17] RR 0.97 (0.90–1.04) RR 1.42 (0.69–2.91) [13]	NS Favors TZP NS NS	Tchero 2018 [15]; NICE 2019 [13]; Peters 2020 [14]; Pratama 2022 [17]
		Graham 2002a [26]	Clinical resolution	RR 0.89 (0.58–1.36) [11]	NS	Selva Olid 2015 [11]; Peters 2020 [14]
		Lipsky 2005a (SIDESTEP) [27]	Clinical resolution Adverse events	RR 1.08 (0.97–1.21) RR 0.76 (0.53–1.09) [11]	NS NS	Selva Olid 2015 [11]; Tchero 2018 [15]; NICE 2019 [13]; Peters 2020 [14]; Pratama 2022 [17]
**Meta-analysis** Ertapenem	TZP	Graham 2002a; Lipsky 2005a	Clinical resolution	RR 1.07 (0.96–1.19) [11]	NS	Selva Olid 2015 [11]
Imipenem	Piperacillin + clindamycin	Bouter 1996 [28]	Clinical resolution Recurrence Adverse events	RR 0.73 (0.24–2.24) RR 7.61 (0.42–139) RR 0.27 (0.09–0.84) [11]	NS NS Favors IMP	Selva Olid 2015 [11]; NICE 2019 [13]
**Penicillins**						
TZP	SAM	Harkless 2005 [29]	Clinical resolution Amputations Adverse events	RR 1.02 (0.86–1.20) RR 0.97 (0.51–1.84) RR 1.14 (0.99–1.32) [11]	NS NS NS	Selva Olid 2015 [11]; Tchero 2018 [15]; NICE 2019 [13]; Peters 2020 [14]; Pratama 2022 [17]
TZP	Imipenem	Saltoglu 2010 [30]	Clinical resolution Amputations Recurrence Adverse events	RR 1.66 (0.84–3.26) RR 0.87 (0.59–1.28) RR 5.31 (0.27–106.46) RR 3.19 (0.95–10.72) [11]	NS NS NS NS	Selva Olid 2015 [11]; Tchero 2018 [15]; NICE 2019 [13]; Peters 2020 [14]; Pratama 2022 [17]
SAM	Imipenem	Grayson 1994 [31]	Clinical resolution Amputation Recurrence Adverse events	RR 0.95 (0.80–1.14) RR 0.85 (0.62–1.15) RR 0.71 (0.42–1.21) RR 1.06 (0.61–1.85) [11]	NS NS NS NS	Selva Olid 2015 [11]; Tchero 2018 [15]; NICE 2019 [13]; Peters 2020 [14]; Tardaguila-Garcia 2021 [16]
AMC	Placebo	Chantelau 1996 [32]	Ulcer healing rate	27.3% vs. 45.5% [15]	Favors placebo	Tchero 2018 [15]
TZP	Ticarcillin-clavulanate	Tan 1993 [33]	Clinical resolution	RR 1.16 (0.59–2.29) [11]	NS	Selva Olid 2015 [11], NICE 2019 [13]
SAM	Cefoxitin	Erstad 1997 [34]	Clinical resolution Amputation Adverse events	RR 0.14 (0.02–1.05) RR 1.00 (0.48–2.08) RR 1.17 (0.49–2.79) [11]	NS NS NS	Selva Olid 2015 [11], NICE 2019 [13], Peters 2020 [14]
**Cephalosporins**						
Ceftriaxone	Cefazolin	Bradsher 1984 [35]	Cure Adverse events	RR 0.84 (0.57–1.24) RR 0.92 (0.48–1.78) [13]	NS NS	NICE 2019 [13]; Peters 2020 [14]
Ceftriaxone + metronidazole	Ticarcillin-clavulanate	Clay 2004 [36]	Cure	RR 1.05 (0.85–1.28) [13]	NS	Selva Olid 2015 [11]; Tchero 2018 [15]; NICE 2019 [13]; Peters 2020 [14];
Ceftriaxone	Fluoroquinolone	Lobmann 2004 [37]	Clinical response	58% vs. 51.1% [14]	NS	Peters 2020 [14]
Ceftriaxone	Levofloxacin + metronidazole	Patil 2016 * [38]	Microbiological cure	58.6% vs. 62.1% [17]	NS	Tchero 2018 [15]; Pratama 2022 [17]
Ceftobiporole	Ceftazidime + vancomycin	Noel 2008a [39]	Clinical resolution	RR 1.05 (0.90–1.23) [11]	NS	Selva Olid 2015 [11]; Peters 2020 [14]
Cefoxitin	Ceftizoxime	Hughes 1987 [40]	Clinical response Adverse events	RR 0.83 (0.60–1.14) RR 1.31 (0.84–2.04) [13]	NS NS	NICE 2019 [13]
Cefoxitin + amdinocillin	Cefoxitin	File 1983 [41]	Clinical response Amputation	RR 1.26 (0.93–1.70) RR 0.53 (0.11–2.56) [13]	NS NS	NICE 2019 [13]
**Others**						
Clindamycin	Cephalexin	Lipsky 1990 [42]	Clinical resolution Ulcer healing Adverse events	RR 1.07 (0.79–1.45) RR 1.20 (0.59–2.46) RR 0.47 (0.04–4.84) [11]	NS NS NS	Selva Olid 2015 [11]; NICE 2019 [13]; Peters 2020 [14]
Daptomycin	Vancomycin or semisynthetic penicillin	Arbeit 2004 [43]	Clinical resolution Adverse effects	RR 0.94 (0.68–1.30) RR 0.61 (0.39–0.94) [11]	NS Favors daptomycin	Selva Olid 2015 [11]
		Lipsky 2005b [44]	Cure (vs. penicillin) Cure (vs. vancomycin)	RR 0.91 (0.62–1.33) RR 1.04 (0.69–1.56) [13]	NS NS	NICE 2019 [13]; Peters 2020 [14]
Tigecycline	Ertapenem +/− vancomycin	Lauf 2014 [45]	Clinical resolution Resolution (OM) Clinical cure Clinical cure (OM) Adverse events	RR 1.09 (1.01–1.18) RR 2.08 (1.27–3.39) [11] RR 0.94 (0.99–1.14) RR 0.69 (0.35–1.32) RR 1.25 (1.13–1.38) [13]	Favors ETP Favors ETP NS NS Favors ETP	Included in all SR
Linezolid	SAM	Lipsky 2004 [46]	Clinical cure Adverse events	RR 1.14 (0.99–1.31) RR 2.66 (1.49–4.73) [13]	NS Favors SAM	Selva Olid 2015 [11]; NICE 2019 [13]; Peters 2020 [14]; Tardaguila-Garcia 2021 [16]

^#^ Outcomes as reported in the systematic reviews; NS: not significant; RR: risk ratio; Diff: difference; TZP: piperacillin–tazobactam; AMC: amoxicillin–clavulanate; SAM: ampicillin–sulbactam; ETP: ertapenem; OM: osteomyelitis; ‡ Labelled as Zhang-Rong 2016 in NICE 2019 [13]; * Labelled as Swati 2016 in Tchero 2018 [15].

## Data Availability

The data presented in this study are available upon request from the corresponding author.

## Appendix A

**Table A1:** Search strategy (last conducted 24 January 2023).

Ovid Medline 1946 to 20 January 2023	exp Diabetic Foot/ exp Foot Ulcer/ (diabet* adj3 ulcer*).mp (diabet* adj3 (foot OR feet)).mp (diabet* adj3 wound*).mp or/1-5 exp Anti-Bacterial Agents/ exp Anti-Infective Agents/ antibiotic*.mp antimicrobial*.mp or/7-10 6 AND 11 limit 12 to (english language and (meta-analysis or “systematic review”))	63 results
Embase via OVID, 1947 to present	exp diabetic foot/ exp foot ulcer/ (diabet* adj3 ulcer*).mp (diabet* adj3 (foot OR feet)).mp (diabet* adj3 wound*).mp or/1-5 exp antibiotic agent/ exp antiinfective agent/ antibiotic*.mp antimicrobial*.mp or/7-10 6 AND 11 Limit 12 to English language, meta-analysis or systematic review	211 results
Cochrane Database of Systematic Reviews, issue 1 of 12 January 2023	MeSH descriptor: [Diabetic Foot] explode all trees [Foot ulcer] explode all trees diabet* NEAR/3 ulcer* diabet* NEAR/3 (foot or feet) diabet* NEAR/3 wound* (#1 OR #2 OR #3 OR #4 OR #5) MeSH descriptor: [Anti-Bacterial Agents] explode all trees MeSH descriptor: [Anti-Infective Agents] explode all trees antibiotic* antimicrobial* (#7 OR #8 OR #9 OR #10) (#6 AND #11) Limits: in Cochrane Reviews and Cochrane Protocols	59 results
CINAHL plus (EBSCOhost)	MH Diabetic Foot MH Foot Ulcer+ diabet* N3 ulcer* diabet* N3 (feet OR foot) diabet* N3 wound* Or/1-5 MH Antibiotics+ MH Antiinfective Agents+ Antibiotic* Antimicrobial* Or/7-10 6 AND 11 with Limiters: English Language; Publication type: Meta Analysis, Systematic Review	38 results
Prospero database of systematic reviews	MeSH DESCRIPTOR Diabetic Foot EXPLODE ALL TREES	128 results

## Appendix B

**Table A2:** Excluded reviews (full-text).

Study	Exclusion Reason
Abolghasemi et al. 2019	Not a systematic review
Awasthi et al. 2021	Not a systematic review
Bartoszko et al. 2018	Protocol only
Esposito et al. 2016	Did not meet inclusion criteria
Game et al. 2016	Did not meet inclusion criteria
Karri V et al. 2016	Not a systematic review
Norman et al. 2016	Did not meet inclusion criteria
Perez-Panero et al. 2019	Did not meet inclusion criteria
Peters et al. 2016	Superseded systematic review
Singh et al. 2021	Not a systematic review (narrative review)
Tchero et al. 2019	Not a systematic review (scoping review)
Urtugrul et al. 2020	Not a systematic review (narrative review)
Vas et al. 2018	Did not meet inclusion criteria
Yazdanapah et al. 2015	Did not meet inclusion criteria
Zhang et al. 2020	Protocol only
